# Organizational Interventions concerning Palliation in Community Palliative Care Services: A Literature Study

**DOI:** 10.5402/2012/769262

**Published:** 2012-08-02

**Authors:** Mette Raunkiær, Helle Timm

**Affiliations:** Danish Knowledge Centre for Palliative Care, Strandboulevarden 47 B, 2100 Copenhagen Ø, Denmark

## Abstract

*Background*. Studies indicate problems between different professional groups working with palliative care and the organisation of palliative home care at nursing homes. The purpose of this study is to examine international experiences and cooperative development initiatives regarding the organisation of community palliative care services. *Method*. The study has been carried out as a literature study based on bibliographic searches in international databases with selected key words. *Results and Conclusion*. The study of the literature identified 19 studies described in 20 articles that relate to development efforts and interventions regarding the organisation of palliative care in communities. Nearly, all of the studies were based on health care professionals' assessments of users (the relatives). However, it is unknown whether or how patients and relatives experience a positive effect of the interventions. The literature study shows that it is a great methodological challenge to complete and evaluate studies concerning organisation and cooperation using methods that make the results useful for others.

## 1. Introduction

After many years of focus on the development of specialised palliative efforts, attention has begun to increase, both in Denmark and internationally, on the need to develop general efforts as well, where many of them take place in communities. This development stems particularly from organisational conditions and competencies [1–3]. 

Studies [4–8] indicate problems between different professional groups working with palliative care and the organisation of palliative home care. The challenges concern, for example, palliative care domains that necessitate specialised competencies, showing respect for the competencies of other health occupations and individuals. Other challenging issues include improved interdisciplinary collaboration and more efficient circulation of information between care settings (e.g., palliative home care teams and/or GPs, district nurses, and hospitals), improved accessibility, continuity and quality of care, and services to patients at the end of life, as well as shared GP and district nurse visits to families at home. Proactive planning from the beginning of the palliative process and clear distribution of tasks are also needed. Studies of nursing homes (NHs) [9, 10] also indicate problems concerning management, cooperation, communication, and shared care between professional groups.

Dying people deserve good care (also meaning good continuity of care). But the above-mentioned publications show that there are ostensibly many aspects of community palliative care services, which can be improved so that the sick and the dying and their relatives achieve comfort and continuity of care. The dying and their relatives need to be seen and treated uniquely by professionals, in other words it is essential to develop care that supports families at the centre of the effort. There is a need to improve and develop organisational structures—for example, professional guidelines and new forms of cooperation that support cooperation between the various professional groups working with community palliative care services and/or cooperation between the basic palliative level and the specialist level. 

The aim of the present literature study is to examine international experiences and cooperative development initiatives regarding the organisation of community palliative care services (in other words, home care, nursing homes, and general medical practice). International experiences in developing competency levels in palliative care in communities have been examined in another study.

## 2. Method

This study is inspired by Polit and Becks [11] methods of how to conduct a literature study and based on bibliographic databases (Pubmed, CINAHL, and PsycInfo) and with selected keywords [11, pages 172–187]. The keywords are described in the Section 2.2. There exist different attitudes whether reviewers should limit sample to published studies or include grey literature, or restrict sample to reports in peer-reviewed journals/publications [11, pages 515–519]. In this literature study, we do not include grey literature because of access to the publications, but we do include not peer-reviewed publications, otherwise the study would represent very few publications. 

### 2.1. Inclusion and Exclusion Criteria for the Literature Search

Inclusion criteria included the following:

- palliation in communities, home care schemes, or nursing homes,
- development initiatives in palliative care in primary health care,
- palliation to adults (≥18 years),
- all diagnoses and selected diagnoses; cancer, dementia, heart and lung diseases,
- reviews and individual studies,
- literature from the years 2000–2010,
- Norwegian, Swedish, Danish, and English language literature.

Exclusion criteria included studies that target:

- organisation and cooperation in the secondary sector exclusively,
- the specialised palliative level exclusively,
- palliation to children (<18 years),
- non-Western material.

### 2.2. The Literature Search Process

The bibliographic databases PubMed/Medline, CINAHL, and PsycInfo were searched from June 2010 to October 2010. 

In PubMed, there were made two searches. The first search was with the keywords “palliative care/org. & admin OR terminal care/org. & admin AND community health services/org. & admin OR primary health care/org. & admin OR community health nursing/org. & admin OR home care services/org. & admin OR nursing homes/org. & admin and neoplasms OR dementia or heart diseases OR lung diseases.” The second search was with the keywords “palliative care/org. & admin OR terminal care/org. & admin AND community health services/org. & admin OR primary health care/org. & admin OR community health nursing/org. & admin OR home care services/org. & admin OR nursing homes/org. & admin AND interinstitutional relations OR interprofessional relations.” 

The search in CINAHL was based on the keywords “palliative care/org. & admin,” “terminal care/org. & admin” with an OR as liaison combined with AND and “community health services OR primary health care AND interinstitutional relations OR interprofessional relations”; “nursing homes AND interinstitutional relations OR interprofessional relations”; “home care services AND interinstitutional relations OR interprofessional relations”; “home care services AND interinstitutional relations OR interprofessional relations.”

In PsycInfo, the keywords were “palliative care,” “terminal care” with OR as liaison combined with AND and “primary health care AND interprofessional relations”; primary health care AND organisation OR administration”; “nursing home AND organisation OR administration”; “nursing home AND interprofessional relations OR interinstitutional relations”; “home care services AND interinstitutional OR interprofessional relations”; “nursing AND interinstitutional OR interprofessional relations”; “nursing AND organisation OR administration.”

The first screening of the publications was based on reading the abstracts (or title if no abstract existed) and in proportion to the inclusion and exclusion criteria. Afterwards, the publications were more carefully read in a full-text version—all time in proportion to the inclusion and exclusion criteria. And at last, the articles were examined based on the analysis questions below. 

### 2.3. Analysis Method

All included studies were analysed with respect to the following analysis questions.

- What country of origin is represented in the studies?
- What kind of study does the publication represent?
- How are the target groups and the professional actors defined?
- How is the development initiative designed, including
  - organizational units included in the development initiatives,
  - the aim,
  - what the development initiative is,
  - which methods are used to evaluate the development initiative,
  - conclusion?

## 3. Results

A total of 19 studies are included (in 20 publications). The preliminary literature search resulted in PubMed: 222 publications; CINAHL: 1397 publications; PsycInfo: 17 publications. After the first screening of the abstracts (or title if no abstract existed) and due to the inclusion and exclusion criteria, the publications were reduced to 215 publications. However, after a deep reading process in full-text versions of the publications, additional 195 publications were excluded due to the previously mentioned exclusion criteria above or for the following reasons: doubles, discussing and describing (i.e., personal cases, personal commentary, or experiences), focus only on symptom management, focus on problem identification and suggested interventions, focus on national and international strategies, and others such as focus on the place of death and consequences for health care policy, and focus on comparisons between patients who receive palliative care and usual care. The remaining 19 studies (20 publications) were analysed based on the analysis questions. 

### 3.1. Country of Origin, Study Type, Target Group, and Professional Actors in the Studies

Only one publication treats development initiatives in Ireland [12], three treat development initiatives in Australia [13], and 16 publications treat development initiatives in the UK. Three studies [13–15] deal with specific patients with heart disease. The other 16 studies have either cancer patients or patient groups with nondefined diagnoses as their targets. Five studies (Table 2) have a particular focus on cooperation and organisation between GPs and/or other care groups. The other 14 studies focus on care personnel. One study is a review [16]. All other studies are individual studies and none are randomised or controlled. The evaluation method is unclear in four studies [13, 15, 17, 18]. 

### 3.2. The Development Initiatives

All studies deal with guidelines and cooperative models across various specialist and basis levels and are related to development efforts and interventions regarding palliation in the following organisation units:

- cooperation between basis and specialised palliative levels and/or other specialised levels,
- cooperation between medical practice and other organisational units in primary health care,
- palliation at nursing homes.

### 3.3. Development Initiatives concerning Cooperation between Basis and Specialised Palliative Levels and/or Other Specialised Levels

Seven studies described development initiatives/interventions (see Table 1). Two recent studies from the UK [16, 18] were concerned with pathways and guidelines referred to as The Golden Standards Framework (GSF), Liverpool Care Pathways (LCP), and Advance Cancer Care Planning (ACP). One of the studies [16] reviewed 15 documents regarding the impact of GSF and showed that GSF improves general practice processes, has a positive impact on control of symptoms, continuity, continued learning, greater understanding of palliative care supporting patients and families, and so forth. Many practices were able to implement the foundation level of the GSF. However, adoption of the higher levels of care appeared more variable. The GSF requires adequate resources. The direct impact on patients and carers is not known. The other study [18] was a model based on GSF, ACP, and LCP as a model of collaborative working. According to community matrons and nurse specialists, the model was helpful in highlighting decisions to ensure that patients and families received optimum care. 

Three out of the seven earlier studies (one from Australia and two from the UK) describe development initiatives connected to palliative care of patients with noncancer diseases such as heart diseases [13–15]. The focus of these studies was on development initiatives regarding nursing care between heart specialists and the palliative specialist level. These initiatives dealt with cooperation between specialist groups such as shared visits to families at home, establishing groups for patients with heart disease, common education for specialist groups, mentorship, visits to each other's practices, and development of palliative thinking in other specialist contexts. Some of the greatest successes involved better communication between specialist groups. One study [14] included patient evaluations of being included in a support group. The intervention was assessed positively.

Two other studies from the UK are concerned with evaluation of two different initiatives: a clinical nurse specialist post [17] and a 72-hour community palliative care nursing service [19]. The evaluation of the new post involved 20% of a clinical nurse specialist's fulltime position being dedicated to working with three palliative care teams. That study concluded that the intervention made progress in improving communication and collaboration between the teams as well as improving recognition and understanding of the constraints under which the teams work. It also provided an opportunity to follow patients and trace their care plan as well as including a more detailed history of the patients' care. Evaluation of the 72-hour community palliative care nursing service was done in connection with audits and questionnaires put to health professionals. That study concluded that the outreach service provided specialist palliative care to patients as they faced the transition between in-patient specialist palliative care and the community setting, particularly providing extra support on discharge from the in-patient unit. The 72-hour service is growing and remains able to respond to patients' needs quickly with expert support.

### 3.4. Development Initiatives concerning the Cooperation between Medical Practice (GPs) and Other Organisational Units

Five studies are concerned with specific initiatives focusing on improving cooperation between GPs and other health professionals (see Table 2). Two previous studies—from the UK and Australia—[20, 21] were concerned with the development and evaluation of a single information sheet, which intended to improve access to clinical information for nurses and doctors providing after-hours community palliative care services and an evaluation of GPs using an out-of-hours protocol. Both development initiatives were evaluated positively.

Three recent studies [22–24] are concerned with the implementation of The Golden Standards Framework (GSF) in the UK. The positive effects of the GSF included an effective approach to a systematic and high-quality service; earlier referral of palliative care patients to district nurses; multidisciplinary team meetings that enabled knowledge sharing, discussion of problems, and keeping colleagues informed. The best functioning teams used a mixture of formal and informal meetings with a relatively nonhierarchical working style between doctors and nurses. Challenges of GFS included an increased nursing workload; fewer or later visits to patients local GSF champions needed to be sustainable, and slow or incomplete adoption. The direct impact on patients and carers is not known. High performance appeared to occur when the implementation of processes promoted by GSF fitted well with the internal environment of practice.

### 3.5. Development Initiatives concerning Palliation at Nursing Homes

Eight publications and seven studies dealt with development initiatives focusing on improvements concerning palliative care at nursing homes (see Table 3). Two earlier studies were concerned with the implementation of specialist services in care homes. One study [12] assessed the current level of input from community-based clinical nurse specialists in palliative care into nursing homes. The assessment of this intervention showed that the main focus of interaction with the nursing homes was on pain and symptom management (physical care) often provided by telephone in connection with patients with cancer diseases. The study concluded that the clinical nurse specialists are ideally placed to provide education and support to nursing homes. The intervention of another study [25] involved the creation of a new specialist palliative nurse post in care homes. The awareness of specialist palliative care services in care homes was established in several ways. The care homes received an updated resource file palliative care service, education in palliative care for newly appointed staff in care homes, two syringe drivers were available to loan, a pain assessment tool was developed, and patients contact with specialist palliative care service was improved. These experiences, based on an unknown foundation of questionnaires and interviews, showed that the care homes felt more able to care for their patients in collaboration with clinical nurse specialists.

A study from Australia [26] examined the process of how residents' end-of-life care (EOLC) wishes were recorded to ensure that the implementation of an advance care plan (ACP) was performed according to the best available evidence. The project had four stages: (1) + (2) interpretation of the five audit criteria related to involving residents (RES) and relatives (REL) in an ACP and providing them with appropriate information of EOLC issues and training the staff regarding an ACP, (3) auditing records of staff and RES, (4) getting research into practice (GRIP): situational analysis, action planning, and action taking to improve compliance with best practice. The GRIP phase showed seven barriers which included deficits related to the knowledge and education of RES, REL, and staff, as well as issues related to administration and documentation, and concerns that any implementation process would not be sustainable. RES and REL expressed a high level of satisfaction with the changes.

Five studies are concerned with the implementation of different guidelines. Two studies are concerned with implementation of the Liverpool Care Pathway (LCP). One study [27] described a pilot project to introduce LCP into care homes in the UK with a view to reducing the number of very ill elderly patients who are transferred to acute trust from care homes. The results showed that LCP had empowered the staff to talk more openly to relatives. They felt able to explain the care, but it was difficult for staff to gauge when to start the pathway. The other study [28] concerned with implementation of LCP to a 150 bed nursing home showed that LCP ensured that the patients received a high standard of palliative care and were allowed to die in the comfort and security of the place they call home. The three recent publications and two studies are concerned with the implementation of The Gold Standards Framework (GSF) in different ways. Two publications [29, 30] described the same study in an attempt to report the impact of implementing The Gold Standards Framework for Care Homes (GSFCH) and an adapted Liverpool Care Pathway for Care Homes (LCP) on seven private nursing homes (NH) in the UK. The study showed that implementing GSFCH and LCP increased the use of do not attempt resuscitation documentation and noted a reduction in unnecessary hospital admissions and hospital deaths. The study also indicated that the staff changed their attitudes about dying; for example, they felt more comfortable in addressing psychosocial and emotional needs, talking to relatives and residents about dying and were more confident in recognising the different stages of the dying process. Another study [31], an evaluation of implementation of GSF in nursing homes in UK, supported the results. The evaluation was based on a pre-post survey design that showed statistically significant increases in the proportion of residents who died in the NHs and those who had an advanced care plan. Crisis admissions to hospital were also significantly reduced.

## 4. Discussion

### 4.1. Design and Methodology of the Studies

These studies are characterised as either being reviews or individual studies. Reviews deal with specific guidelines (GSF). One of the major current challenges in the field of heath care is to develop and promote evidence-based practice [32]. In health care science, evidence-based practice or validity is connected to controlled and preferably anonymised randomized studies. However, when the starting point is organisational development in a local context, it can be both difficult and possibly also meaningless to carry out controlled studies. It can be difficult to generalise results from one context with particular economic, organisational, professional, cultural, and possibly relational and individual conditions, to others. 

The later studies appear more systematic and transparent regarding methods of intervention and assessment, while the earlier studies appear less clear in these areas. It is difficult to judge the effect of a development initiative or intervention when the evaluation designs are only described and not controlled in relation to “before and after” observations or control groups. In addition, the results of the interventions are almost completely built on the experiences of the professionals and only in a few cases, not systematically, include the judgements of patients or relatives. As a result, we do not know how these interventions change practice seen from the user's perspective.

### 4.2. Principal Findings

16 out of the 19 publications dealt with studies in the UK. This is noteworthy since countries like Australia, Ireland, Norway, Sweden, and Denmark have national strategies (although different in character) for palliative efforts on the whole or in part. This can be an expression of the fact that the UK is one of the countries with the longest palliative tradition [33, 34], which might mean that new development initiatives are first developed and tried out in an English context and then inspire other countries. However, there is a great difference between various western countries and their welfare systems. This includes the organisation of the health care system, financing of palliative payments (percentage of own payment versus tax-based financing), education of professionals, and so forth. Therefore attempts to transfer experiences and interventions from one national context to another can be uncertain, difficult, and debatable. 

 The study indicates that interventions have changed character during the period from 2000 to 2010. At the start of that period, there was a particular focus on cooperative models between the basis and specialised levels of palliation, trials of diagrams attempting to improve multidisciplinary cooperation and communication regarding families (e.g., [20, 21]), and trials of special intervention at discharge (72 hr special palliative service) [17], and so forth. In later years (e.g., [18, 30]), the focus has been on description, implementation, and evaluation of various guidelines, most recently on The Gold Standards Framework that specifically addresses palliative care in primary health care. This might be because the UK has a well-developed structure for quality control of clinical guidelines where, since 1999, they have had one of the world's leading units, the National Institute for Health and Clinical Excellence, NICE, which is part of the National Health Care Service (NHS) [35]. 

The effect of the guidelines (GSF, LCP, etc.) is largely seen solely based on the assessment of professionals, which shows that implementation of guidelines has had a positive impact on multidisciplinary cooperation, communication, and so forth. Only one of the studies [29] includes users (here relatives) assessments. Experience from other studies shows that interventions do not necessarily change practice [36–38]. In other words, the implementation of guidelines alone cannot be considered a guarantee of improved practice despite positive professional assessment, unless the evaluation of development initiatives and interventions also includes the judgement of patients, residents, and relatives. 

Only three publications out of 19 dealt with experiences with organisation and cooperation in relation to specific diagnostic groups—heart disease [13–15]. That might indicate that there is a need for the development of palliative efforts for other diagnostic groups such as dementia syndromes, lung disease, and so forth. 

Studies show (e.g., [4, 39]) problems with cooperation between GPs and nursing care staff in connection with palliative care when patients need care from both the primary and secondary sectors. However, only five out of 18 studies (Table 3) have a more direct focus on cooperation between GPs and nursing staff and only one study [19] addressed the connection involving the transfer of a patient between different settings. This indicates a need for development initiatives in these areas.

### 4.3. Strengths and Weaknesses of This Literature Study

This literature study covers only 19 studies, described in 20 publications. It could be that this is an indication that an intervention focusing on cooperation in community palliative care services is a marginal area. On the other hand, it might also be assumed that some of the development work being done is not being transmitted to academic fora. Primary community initiatives do not have a well-developed tradition for research and publication.

An expanded time period, for example, 1995–2010, and searches of several more databases might have produced more publications. In addition, non-Western studies and studies not written in one of the Scandinavian languages or English have been excluded here and might have reduced the number of studies and insights in the variation of development initiatives within community palliative basis care in non-Western countries.

The literature study is built on selected keywords. This does not exclude the possibility that the use of other key words could have led to even more relevant studies. 

A secondary purpose of the study has been to include experiences from completed interventions in the primary sector. Those studies which exclusively cover problem areas, contain suggestions for interventions, or are characterised by suggestions and strategies have been excluded. That has reduced the number of publications considerably. Additionally, this literature study includes only studies which describe interventions at the basis level of palliation; in other words, studies which only focus on various types of specialist levels have been excluded. This may have reduced the number of included publications since countries have different organisations and understandings of basis and specialist palliative levels. An example with respect to Denmark and the UK is concerned with the Macmillan nurses (MN). MNs are a kind of specialist nurse in the UK, though this is not part of the Danish understanding of specialised palliative institutions (i.e., hospices and palliative teams). However, MNs are often involved in palliative processes in the primary sector in the UK as a type of specialist. This type of specialised nursing does not exist in Denmark and the palliative basis level nursing care consists solely of home care nursing. It is possible that if specialist nurses, MNs, had been defined as part of the palliative basis level in this literature study, more studies would have been included. 

## 5. Conclusion

The literature study shows few and sparse studies with interventions concerning cooperative models within community palliative care services. It seems characteristic that they deal with individual studies and not controlled studies. Nearly all of the studies were based on health care professionals' assessments of users (the relatives). However, it is unknown whether or how patients and relatives feel a positive effect of the interventions. Additionally, the literature study shows that there is a lack of studies with focus on development initiatives concerning particular diagnosis groups (i.e., dementia, and KOL) cooperating with GPs and nursing personnel and the related palliative care when patients have needs that cross the primary and secondary sectors. Last, but not least, it is a great methodological challenge to complete and evaluate studies concerning organisation and cooperation using methods that make the results useful for others.

## Figures and Tables

**Table 1:** Development initiatives regarding cooperation between the basis and specialised palliative levels and/or other specialised levels.

Author, year, and place	The aim	Development initiatives/interventions	Methods of the evaluation	Conclusion
Davidson et al., 2004 [13], Australia	To describe the development of a model of an integrated, consultative, palliative care approach within a comprehensive chronic heart failure (HF) communication-focused disease management program.	The model has four areas: diagnosis and secondary prevention; rehabilitation and promotion of self-care strategies; reinforcement, monitoring, and community coordination of care; collaborative palliative care support of families. Education and training in end-of-life physical symptoms and emotional and ethical issues; palliative care approach.	Unclear.	Communication between teams was improved. Division of GP has been pivotal in developing the model by endorsement of the model and provision of educational activities and dissemination of communication.
Plummer and Hearnshaw, 2006 [19], UK	To describe and evaluate short-term specialist palliative care at home.	A 72-hour community palliative care nursing service to patients moving between in-patient and community care includes 24-hour care; responds to specialist palliative care needs within the community setting; provides short periods of specialist care; allows for a preadmission assessment; provides a short period of intensive support to try and prevents admission; facilitates access to 24-hour inpatient beds; enables a rapid discharge from hospice/hospital setting; enables a patient to die at home.	In-house audit examined records of all patients referred during the first year (*n* = 61); and 55 questionnaires to healthcare professionals in the locality, 27 returned (respond rate = 49%).	The service responded to patients' needs quickly with expert support.
Daley et al., 2006 [14], UK	To describe the evolution of joint working between heart failure specialists (HFNSs) and specialist palliative care services in Bradford.	HFNS attended the community palliative care team's regular multidisciplinary team meetings (MDTs); formal education by the palliative care service and vice versa; practice-based education for primary care staff by HFNSs and consultant. Collaboration over patient care: telephone advice; joint case discussion and visits with a Macmillan nurse; medical assessment at a hospice-based outpatient clinic; hospice admission for symptom control or terminal care. A Heart Failure Support Group (HFSG) for patients and relatives.	Data collection, audit, and evaluation performed by the Heart Failure Nurse Specialist (HFNS) and palliative care service: a shared electronic clinical record system; recorded key information on a database; data from the patients' paper records; qualitative data on 15 patients' experiences of the support group.	The HFSGs help patients to cope in key areas such as: physical, psychological, and social isolation; loss of self-esteem and sel-worth; generating hope and purpose. The HFNSs can function as key workers, providing support throughout the illness and maintaining continuity of care. Few patients have needed direct care from the specialist care service.
Dawson, 2007 [17], UK	To evaluate the impact of a new post, where 20% of a clinical nurse specialist's (CNS) full-time post was dedicated to working between three palliative care teams in Manchester.	The time was used on visiting patients on the wards; nurse specialists accompanied the CNS in patients' homes; contributing to an audit tool; joint teaching sessions; developing palliative care concerning end stage renal failure; providing a link between patients' community and hospital to exchange information; initiate a referral.	Unclear.	Progress in improving communication and collaboration between the teams was noticed. Opportunity to follow patients. More detailed history of the patients' care. Further recommendations: monthly and bimonthly interprofessional meetings (face-to-face dialogue); shadowing colleagues in practice; joint educations sessions.
Pooler et al., 2007 [15], UK	To discuss the lack of equity of palliative care for patients with heart failure and what a hospice Macmillan clinical nurse specialist (MCNS) team sought to achieve by working collaboratively with their community heart failure nurse specialist (HFNS) colleagues.	Education of HFNS and MCNS. The referral criteria as developed by Merseyside and Cheshire Specialist Palliative Care and Cardiac Clinical Networks were adopted. Standard referral forms were used to monitor referrals to the service and track outcome. HFNSs remain key worker, using the MCNS as a resource. HNFS and MCNS undertook joint assessment visits at the patients.	Unclear.	Open and honest communication between professionals and the families. Joint visits ensured that that HFNS and MCNS worked outside their own professional competence and enabled new learning to take place.
Alsop, 2010 [18], UK	To support community matrons in their care of patients at the end of life through the creation of a new model of collaborative working.	Pathways to clarify decision making were developed into a guide/model for use by health or social care/professional care for any patient irrespective of diagnosis. The model was based on the Gold Standards Framework (GSF), Advance Cancer Planning (ACP), and Liverpool Care Pathways (LCP).	Unclear.	The model helped the community matrons and nurse specialists to ensure that patients and families received optimum care and contributed to understanding of the role of palliative care in supporting patients and families.
Shaw et al., 2010 [16], UK	To examine/review the impact of the GSF on general practice systems and procedures in primary care; GSF providers (i.e., the healthcare practitioners delivering the GSF, GSF users (i.e., patients and carers).	The GSF improves general practice processes, coworking, and the quality of palliative care, but can be undermined by lack of shared commitment. GSF had a positive impact on control of symptoms, continuity, continued learning, caregiver support, and the caregiver in the dying phase. Many practices are able to implement the foundation level of the GSF. However, adoption of the higher levels of care is more variable. The GSF requires adequate resources. The direct impact on patients and carers is not known.		GSF has considerable potential to improve end-of-life care, but further work is needed to support uptake and consistency of implementation. Additional evidence about patient and carer outcomes will add to existing insights.

**Table 2:** Development initiatives concerning the cooperation between medical practices and other organisational units.

Author, year, and place	The aim	Development initiatives/interventions	Methods of evaluation	Conclusion
King et al., 2003 [21], UK	To examine the experiences of primary care practitioners in using the out-of-hours protocol, and their perceptions of its effectiveness.	The protocol was organised around four priority areas: communication; care support; specialist medical advice; drugs and equipment.	Four group interviews with 20 district nurses and individual telephone interviews with 15 GPs.	The protocol had facilitated better communication between in- and out-of-hours services; promoted a more anticipatory approach to care; better access to drugs through the Bearder bags. Recommendations for future development: carer support, regularly updating the forms of the scheme.
Brumley et al., 2006 [20], Australia	To improve access to clinical information for nurses and doctors providing after hours community palliative care in a regional Australian setting.	Development of a single information sheet on the community palliative care service computers with: medical history; treatments, current status; up-to-date medications list; progress notes; risks and problems; symptom control; contact information; doctors' letters; expectations of care.	Palliative care nurses and GPs surveys and focus group feedback; the number of accurate predictions of unstable palliative care patients that resulted in call-outs after hours; patient satisfaction survey following after hours service.	Information would have been useful if GPs had been contacted about patients after hours. The palliative care nurses felt confident with the assessments being more professional in their practice with other medical colleagues, were able to provide current information.
Munday et al., 2007 [23], UK	To explore the effectiveness and sustainability of the implementation of The Gold Standards Framework (GSF) at practice level.	Implementation of GSF at practice level in 15 practices from three areas in the UK which had commenced GSF implementation between March 2003 and September 2004.	Interviews and observational data with 15 practices participating in GSF. Semi-structured interviews (total 45) with GPs, community nurses, and practice managers. Supplied by observation of practice meetings and systems, to provide contextual insights. Analysis: thematic matrix approach and comparison between practices.	High performing practice of GSF procedures implied clear, shared purpose for palliative care with effective communication. Few performing practices demonstrated little utilization of basic GSF processes and deficiencies in interprofessional communication.
Mahmood-Yousuf et al., 2008 [22], UK	To investigate the extent to which the framework (Gold Standards Framework (GSF)) influences interprofessional relationships and communication, and to compare GPs' and nurses' experiences.	Implementation of GSF at practice level in 15 practices from three areas in the UK, which had commenced GSF implementation between March 2003 and September 2004.	15 practices participated. 38 semi-structured interviews with GPs, district nurses, Macmillan nurses, and framework facilitators.	Adoption of GSF resulted in earlier referral of palliative care patients to district nurses. Multidisciplinary team meetings enabled communication for sharing knowledge, discussing management problems, but arranging and maintaining meetings were often problematic. Nurses in particular valued formal meetings while GPs generally preferred informal and ad hoc dialogues. The best functioning teams used a mixture of formal and informal meetings with a relatively nonhierarchical working style.
Walshe et al., 2008 [24], UK	To present data on the anticipation and adoption of the GSF.	Implementation of the GSF within three Primary Care Trusts in North West England.	47 interviews with generalist and specialist palliative and primary care professionals (district nurses, GP, allied health professionals, managers commissioners, specialist palliative care nurses, doctors, and allied health professionals).	Positive benefits to professionals included improved interprofessional communication and anticipatory prescribing. Negative aspects were increased nursing workload and the possibility of fewer or later visits to patients. GSF needed local champions to be sustainable. Slow or incomplete adoption was reported.

**Table 3:** Development initiatives/interventions concerning nursing homes.

Author, year, and place	The aim	Development initiatives/interventions	Methods of evaluation	Conclusion
Ling, 2005 [12], Ireland	To assess the current level of input from community-based clinical nurse specialists (CNSs) in palliative care into nursing homes in Ireland.	Telephone contact with nursing homes on pain and symptom management. The majority of nurses were involved exclusively in care of patients with cancer, although 40% of respondents cared for patients with nonmalignant diseases.	A national survey of all community-based CNS, 116 questionnaires, and 65 responses.	CNS in palliative care in nursing homes focuses on physical care. CNS specialists are ideally placed to provide education and support to nursing homes.
Edwards and Hirst, 2005 [25], UK	The intervention (new specialist nurse post) was to improve the accessibility and availability of generalist and specialist care and palliative care (PC) resources in the district and to ensure high-quality end-of-life care for patients in care homes (CH) in Wakefield.	All CHs received an updated resource file for PCS. Education: an “Introduction to PC” training session was developed for newly appointed staff in the CH about syringe drivers and pain assessment. Patient contact with the CNS.	Questionnaire to NHs (number is unclear).	The CHs were appreciative of the support and felt more able to care for their patients. An increase in post for further 22.5 hours a week.
Duffy and Woodland, 2006 [27], UK	To describe a pilot project to introduce the Liverpool Care Pathway (LCP) into care homes local to the Queen Mary's Sidcup NHS Trust with a view to reducing the number of very ill elderly patients who are transferred to acute trust from care homes.	Implementation of LCP at a care home. Two flow charts were designed with a view to guide the staff. Audit pack. Meetings with the GPs and district nurses. Resource files for each of the units were produced.	Unclear, but involved audits, registration of deaths in home or hospital before and after implementation of LCP, feedback from the involved professionals including GPs.	LCP had empowered the staff to talk more openly to relatives and they felt more familiar with the paperwork; possibility to prepare ahead; ask the GP to prescribe drugs in advance. But it was difficult for staff to gauge when to start the pathway. GPs felt that overall the implementation had gone well.
Mathews and Finch, 2006 [28], UK	To outline a pilot project to introduce LCP to a 150-beds nursing home.	Implementation of the LCP included discussion with the local GPs, information to the local out-of-hours chemist, and ambulance service. Education of key professionals. All trained nursing staff received three hours of palliative care education.	Unclear, but involved audits of 10 patients on the LCP and a reflection group.	The audit showed improvement of documentation and assessment of the key symptoms they experienced. LCP ensured that the patients received a high standard of palliative care and were allowed to die in the comfort and security of the place they call home.
Fernandes, 2008 [26], Australia	To examine the process of how residents' end-of-life care (EOLC) wishes are recorded and to ensure that the implementation of an advance care plan (ACP) is performed according to the best available evidence.	Implement and ensure the implementation of ACP. Developed audit criteria: (1) documented evidence that the RES has been involved in ACP, (2) documented evidence that RELs have had the opportunity to be involved in an ACP, (3) staff who complete ACP have received training in this area, (4) staff who implement ACP have received education regarding EOLC issues, (5) documented evidence that the relatives have received education regarding changes in the end-of-life phase.	Pre- and postimplementation audits of 100 residents' documentation and 20 staff to determine compliance following the second stage.	Preimplementation audit indicated poor compliance with best practice, less than 50%. Compliance increased for all criteria after implementation of the process, ranging from 77% to 100 %. The evaluation showed seven barriers, which included deficits related to the knowledge and education of RES, REL, and staff, and issues related to administration and documentation, and concerns that any implementation process would not be sustainable. RES and REL expressed a high level of satisfaction with the changes.
Badger et al., 2009 [31], UK	To evaluate the impact of the introduction of the Gold Standards Framework for care homes (GSFNH) in nursing homes in England.	The research framework was based on a modified action research approach. Implementation of the GSFNH (phase 1) included introducing the organisational tool, the GSFCH, support to homes from a local GSFCH facilitator, support by the development team, a helpline and conference calls, training for care home staff, and support of NH managers.	Pre- and postsurvey. The 95 NHs were invited to participate in the evaluation. NHs completed a baseline survey of care provision and an audit of five most recent resident deaths. The survey and audit were repeated post programme completion. 49 homes returned completed pre- and post- surveys, 44 returned pre- and postdata on deaths.	Statistically significant increases in the proportion of residents who died in the NHs and those who had an Advanced Care Plan. Crisis admissions to hospital were significantly reduced.
Hockley et al., 2010 [30], UK	To report the impact of implementing The Gold Standard Framework for Care Homes (GSFCH) and an adapted Liverpool Care Pathway for Care Homes (LCP) at seven private nursing homes (NH).	Implementation of GSFCH and LCP included workshops and a course, visits to each NH every 10–14 days by the facilitators.	Quantitative data from all clinical notes on deceased residents from two cohorts: those who had died a year previous to the project and those who had died during/following the implementation of the GSFCH/LCP. Staff audits: a sheet with 50 statements was sent to all trained nurses and carers who had been at the NHs for the duration of the project.	There was a highly statistically significant increase in the use of do not attempt Resuscitation (DNAR) documentation, Advance Care Planning and use of the LCP. A reduction in unnecessary hospital admissions and a reduction in hospital deaths from 15% deaths before study to 8% deaths after study. The staff felt more comfortable in addressing psychosocial and emotional needs; in talking to relatives and residents about dying; more confident in recognizing the different stages of the dying process.
(Watson et al., 2010) [29], UK (Same study Hockley et al., 2010) [30]	To report the impact of implementing The Gold Standard Framework for Care Homes (GSFCH) and an adapted Liverpool Care Pathway for care homes (LCP) at seven private nursing homes (NH).	Implementation of GSFCH and LCP.	Qualitative interviews with 22 bereaved relatives before, 14 bereaved relatives, and six care home managers after implementation of the GSFCH and LCP into seven care homes.	Care home staff changed their attitudes about dying. This enabled more informed end-of-life decision making involving REL, staff, and GPs. REL talked less about poor care. Improvements in care of the dying following implementation of both tools.

## References

[1] Department of Health (2008). *End of Life Care Strategy: Promoting High Quality Care for All Adults at the End for Life*.

[2] Radbruch L, Payne S (2009). White paper on standards and norms for hospice and palliative care in Europe: part 1. *European Journal of Palliative Care*.

[3] Radbruch L, Payne S (2010). White paper on standards and norms for hospice and palliative care in Europe: part 2. *European Journal of Palliative Care*.

[4] Neergaard MA, Olesen F, Jensen AB, Søndergaard J (2010). Shared care in basic level palliative home care: organizational and interpersonal challenges. *Journal of Palliative Medicine*.

[5] Morin D, Saint-Laurent L, Bresse MP, Dallaire C, Fillion L (2007). The benefits of a palliative care network: a case study in Quebec, Canada. *International Journal of Palliative Nursing*.

[6] Goldschmidt D, Groenvold M, Johnsen AT, Stromgren AS, Krasnik A, Schmidt L (2005). Cooperating with a palliative home-care team: expectations and evaluations of GPs and district nurses. *Palliative Medicine*.

[7] Sullivan KA, McLaughlin D, Hasson F (2005). Exploring district nurses’experience of a hospice at home service. *International Journal of Palliative Nursing*.

[8] Henriksen HT, Riis J, Leikersfeldt G (2002). Den terminale kræftpatient i hjemmet: erfaringer fra et hospicebaseret palliativt team. *Ugeskrift for Læger*.

[9] Raunkiær M, Timm H (2010). Development of palliative care in nursing homes: evaluation of a Danish project. *International Journal of Palliative Nursing*.

[10] Raunkiær M (2010). Forestillinger og erfaringer om døden på plejehjem. *Klinisk Sygepleje*.

[11] Polit DF, Beck CT (2010). *Nursing Research: Appraising Evidence for Nursing Practice*.

[12] Ling J (2005). Palliative care in Irish nursing homes: the work of community clinical nurse specialists. *International Journal of Palliative Nursing*.

[13] Davidson PM, Paull G, Introna K (2004). Integrated, collaborative palliative care in heart failure: the St. George Heart Failure Service experience 1999–2002. *The Journal of Cardiovascular Nursing*.

[14] Daley A, Matthews C, Williams A (2006). Heart failure and palliative care services working in partnership: report of a new model of care. *Palliative Medicine*.

[15] Pooler J, Yates A, Ellison S (2007). Caring for patients dying at home from heart failure: a new way of working. *International Journal of Palliative Nursing*.

[16] Shaw KL, Clifford C, Thomas K, Meehan H (2010). Improving end-of-life care: a critical review of the Gold Standards Framework in primary care. *Palliative Medicine*.

[17] Dawson S (2007). Interprofessional working: communication, collaboration... perspiration!. *International Journal of Palliative Nursing*.

[18] Alsop A (2010). Collaborative working in end-of-life care: developing a guide for health and social care professionals. *International Journal of Palliative Nursing*.

[19] Plummer S, Hearnshaw C (2006). Reviewing a new model for delivering short-term specialist palliative care at home. *International Journal of Palliative Nursing*.

[20] Brumley D, Fisher J, Robinson H, Ashby M (2006). Improving access to clinical information in after hours community palliative care. *Australian Journal of Advanced Nursing*.

[21] King N, Thomas K, Bell D (2003). An out-of-hours protocol for community palliative care: practitioners’perspectives. *International Journal of Palliative Nursing*.

[22] Mahmood-Yousuf K, Munday D, King N, Dale J (2008). Interprofessional relationships and communication in primary palliative care: impact of the Gold Standards Framework. *British Journal of General Practice*.

[23] Munday D, Mahmood K, Dale J, King N (2007). Facilitating good process in primary palliative care: does the Gold Standards Framework enable quality performance?. *Family Practice*.

[24] Walshe C, Caress A, Chew-Graham C, Todd C (2008). Implementation and impact of the Gold Standards Framework in community palliative care: a qualitative study of three primary care trusts. *Palliative Medicine*.

[25] Edwards A, Hirst P (2005). Supporting palliative care in care homes: the way forward. *European Journal of Palliative Care*.

[26] Fernandes G (2008). Implementaiton of best practice in advance care planning in an “ageing in place” aged care facility. *International Journal of Evidence-Based Healthcare*.

[27] Duffy A, Woodland C (2006). Introducing the Liverpool Care Pathway into nursing homes. *Nursing Older People*.

[28] Mathews K, Finch J (2006). Using the Liverpool Care Pathway in a nursing home. *Nursing Times*.

[29] Watson J, Hockley J, Murray SA (2010). Evaluating effectiveness of the CSFCH and LCP in care homes. *End of Life Care*.

[30] Hockley J, Watson J, Oxenham D, Murray SA (2010). The integrated implementation of two end-of-life care tools in nursing care homes in the UK: an in-depth evaluation. *Palliative Medicine*.

[31] Badger F, Clifford C, Hewison A, Thomas K (2009). An evaluation of the implementation of a programme to improve end-of-life care in nursing homes. *Palliative Medicine*.

[32] Mattila E, Leino K, Paavilainen E, Aastedt-Kurki P (2009). Nursing intervention studies on patients and family members: a systematic literature review. *Scandinavian Journal of Caring Sciences*.

[33] Hockley J, Clark D (2008). *Palliative Care for Older People in Care Homes*.

[34] Addington-Hall JM, Higginson IJ (2005). *Palliative Care for Non-Cancer Patients*.

[35] http://www.nice.org.uk/.

[36] Emilsson UM (2008). Identity and relationships: on understanding social work with older people suffering from dementia. *Journal of Social Work Practice*.

[37] Emilsson UM (2006). Supervision as pedagogy and support in the Swedish eldercare—a developmental project. *Journal of Gerontological Social Work*.

[38] Emilsson MU (2004). *Handledning och lärande i äldreomsorgens vardag*.

[39] Goldschmidt D (2006). *Evaluation of palliative home care: views of patients, carers, general practitioners and district nurses [Ph.D. thesis]*.

